# Reduced left atrial cardiomyocyte PITX2 and elevated circulating BMP10 predict atrial fibrillation after ablation

**DOI:** 10.1172/jci.insight.139179

**Published:** 2020-08-20

**Authors:** Jasmeet S. Reyat, Winnie Chua, Victor R. Cardoso, Anika Witten, Peter M. Kastner, S. Nashitha Kabir, Moritz F. Sinner, Robin Wesselink, Andrew P. Holmes, Davor Pavlovic, Monika Stoll, Stefan Kääb, Georgios V. Gkoutos, Joris R. de Groot, Paulus Kirchhof, Larissa Fabritz

**Affiliations:** 1 Institute of Cardiovascular Sciences and; 2 Institute of Cancer and Genomics Sciences, College of Medical and Dental Sciences, Medical School, University of Birmingham, Birmingham, United Kingdom.; 3 Institute of Human Genetics, Genetic Epidemiology, WWU Münster, Münster, Germany.; 4 Roche Diagnostics GmbH, Penzberg, Germany.; 5 Department of Medicine I, University Hospital Munich, Ludwig Maximilian University of Munich (LMU), Munich, Germany.; 6 German Centre for Cardiovascular Research (DZHK), partner site Munich Heart Alliance, Munich, Germany.; 7 Department of Cardiology, Amsterdam University Medical Center (UMC), University of Amsterdam, Heart Center, Amsterdam, Netherlands.; 8 Cardiovascular Research Institute Maastricht, Genetic Epidemiology and Statistical Genetics, Maastricht University, Maastricht, Netherlands.; 9 Atrial Fibrillation NETwork (AFNET), Münster, Germany.; 10 Health Data Research Midlands, Birmingham, United Kingdom.; 11 Department of Cardiology, University Hospitals Birmingham (UHB) and Sandwell and West Birmingham (SWBH) NHS Trusts, Birmingham, United Kingdom.; 12 University Heart and Vascular Center, Universitätsklinikum Hamburg-Eppendorf (UKE), Hamburg, Germany.; 13 German Center for Cardiovascular Research, partner site Hamburg/Kiel/Lübeck, Germany.

**Keywords:** Cardiology, Arrhythmias

## Abstract

**BACKGROUND:**

Genomic and experimental studies suggest a role for *PITX2* in atrial fibrillation (AF). To assess if this association is relevant for recurrent AF in patients, we tested whether left atrial *PITX2* affects recurrent AF after AF ablation.

**METHODS:**

mRNA concentrations of *PITX2* and its cardiac isoform, *PITX2c*, were quantified in left atrial appendages (LAAs) from patients undergoing thoracoscopic AF ablation, either in whole LAA tissue (*n* = 83) or in LAA cardiomyocytes (*n* = 52), and combined with clinical parameters to predict AF recurrence. Literature suggests that BMP10 is a *PITX2*-repressed, atrial-specific, secreted protein. BMP10 plasma concentrations were combined with 11 cardiovascular biomarkers and clinical parameters to predict recurrent AF after catheter ablation in 359 patients.

**RESULTS:**

Reduced concentrations of cardiomyocyte *PITX2*, but not whole LAA tissue *PITX2*, were associated with AF recurrence after thoracoscopic AF ablation (16% decreased recurrence per 2^–(ΔΔCt)^ increase in *PITX2*). RNA sequencing, quantitative PCR, and Western blotting confirmed that *BMP10* is one of the most *PITX2*-repressed atrial genes. Left atrial size (HR per mm increase [95% CI], 1.055 [1.028, 1.082]); nonparoxysmal AF (HR 1.672 [1.206, 2.318]), and elevated BMP10 (HR 1.339 [CI 1.159, 1.546] per quartile increase) were predictive of recurrent AF. BMP10 outperformed 11 other cardiovascular biomarkers in predicting recurrent AF.

**CONCLUSIONS:**

Reduced left atrial cardiomyocyte *PITX2* and elevated plasma concentrations of the *PITX2*-repressed, secreted atrial protein BMP10 identify patients at risk of recurrent AF after ablation.

**TRIAL REGISTRATION:**

ClinicalTrials.gov NCT01091389, NL50069.018.14, Dutch National Registry of Clinical Research Projects EK494-16.

**FUNDING:**

British Heart Foundation, European Union (H2020), Leducq Foundation.

## Abstract 2

Building on genomic and experimental findings, low PITX2 in left atrial cardiomyocytes and high BMP10 in plasma predict recurrent atrial fibrillation in patients after ablation.

## Graphical abstract

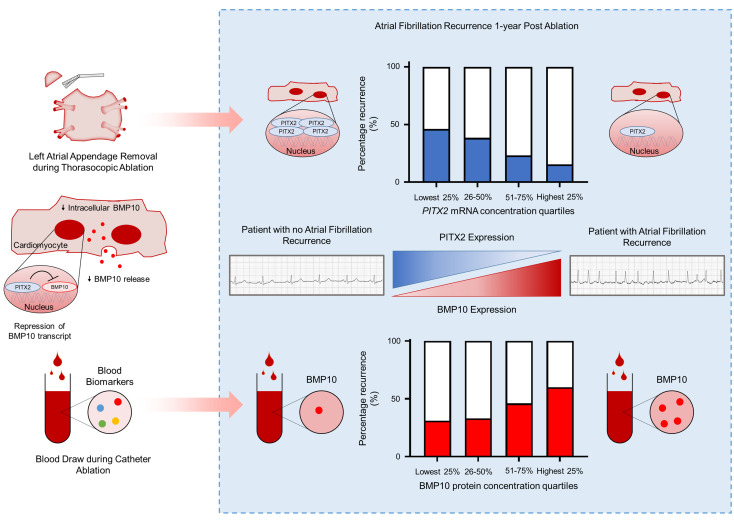

## Introduction

Since it was first described in an Icelandic population (1), genome-wide association studies have consistently identified several common gene variants in a small region on chromosome 4q25 that are strongly associated with atrial fibrillation (AF) (2). These common gene variants are also associated with recurrent AF after AF ablation (3–6). *PITX2*, the gene located closest to this region, encodes for a transcriptional factor that regulates left-right asymmetry in the heart and other organs during development (7). In addition, *Pitx2* suppresses left atrial automaticity and formation of “sinus node–like structures” in the left atrium (8) and contributes to formation of the pulmonary vein myocardium (9). In the adult heart, *PITX2* expression remains largely restricted to the left atrium, where the cardiac isoform *PITX2c* is found (10). In fact, *Pitx2* emerges as one of the most differentially expressed left atrium–specific genes in mice (10, 11) and in patients (10), while the *Pitx2*-regulated gene *Bmp10* is confined to right atrium (11, 12). In mice, reducing *Pitx2* or *Pitx2c* creates a predisposition to AF without marked structural changes in the atria (10, 13–15) via shortened atrial repolarization (13, 15), a more depolarized resting membrane potential (15), and potentially via disrupted calcium handling (14, 16). Gene expression analyses highlight that *Pitx2c* controls expression of ion channels and desmosomal genes (12, 16, 17). These alterations in gene expression are brought about by an altered balance in the atrial network of transcription factors (18, 19). Taken together, these findings suggest that reduced left atrial *PITX2* could predispose patients to recurrent AF after AF ablation. Due to limited access to left atrial tissue in patients whose primary condition is AF, and due to the lack of a more widely accessible marker for left atrial *PITX2*, it remains unclear whether this biologically plausible association exists in patients.

To assess the role of left atrial *PITX2* in recurrent AF in patients, we examined whether left atrial *PITX2* is associated with recurrent AF in patients undergoing thoracoscopic AF ablation. As left atrial *PITX2* concentrations cannot be easily measured in patients, we also sought to identify a blood biomarker that is regulated by left atrial *PITX2*. Based on a literature review, a gene expression screen, and validation through molecular biology experiments in mice with reduced *Pitx2*, we found that genetic reduction of *Pitx2* prominently increases *Bmp10* in the left atrium. As BMP10 is a heart-restricted, secreted protein, we subsequently quantified BMP10 plasma concentrations in patients undergoing AF ablation as a surrogate for left atrial *PITX*2 and assessed its value in predicting recurrent AF after catheter ablation.

## Results

### Whole tissue left atrial PITX2 is uninformative for the prediction of recurrent AF after thoracoscopic ablation.

**Figure 1:**
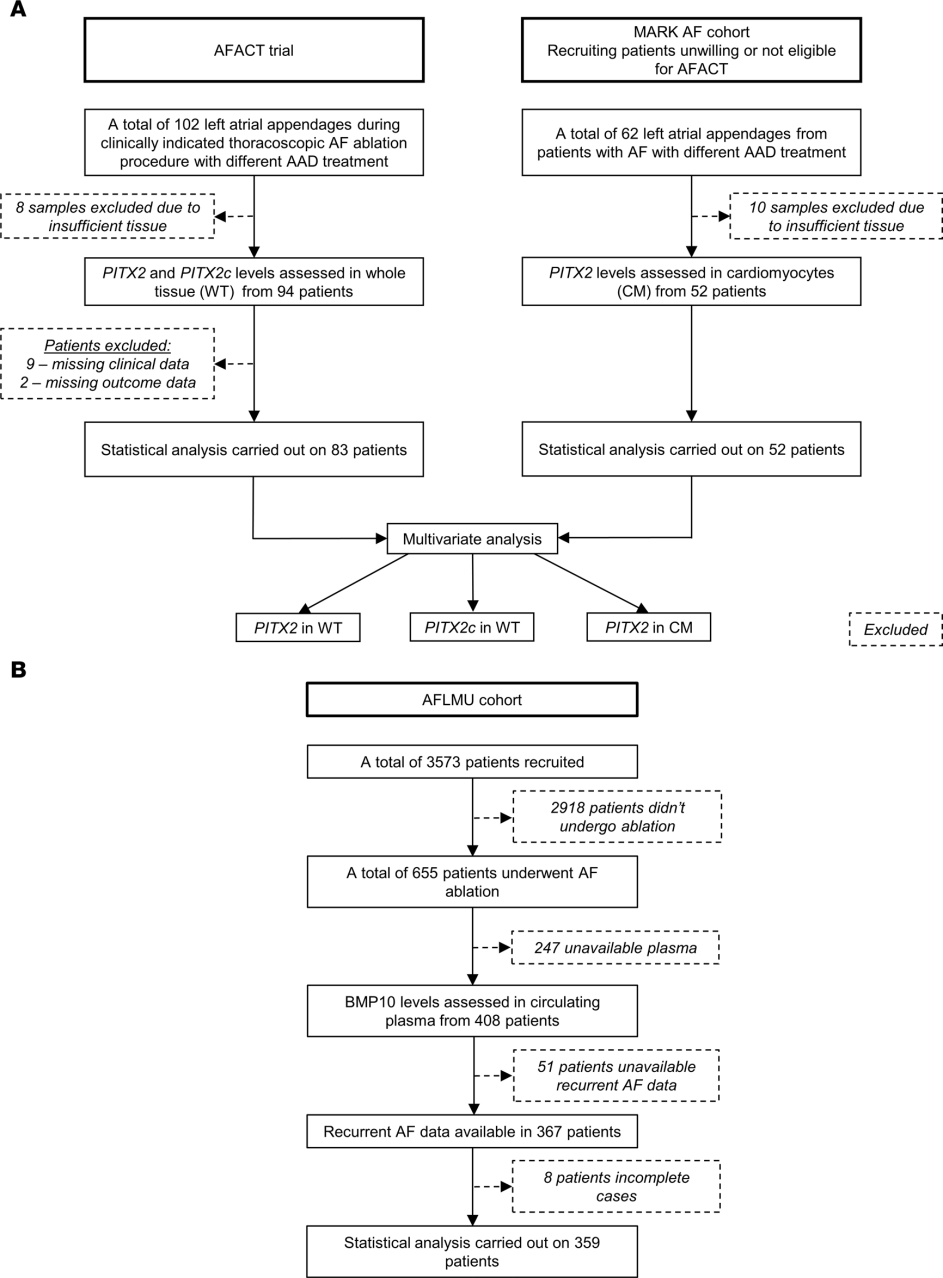
Flow diagram of patients included in the study, and analysis plan.

**Figure 2:**
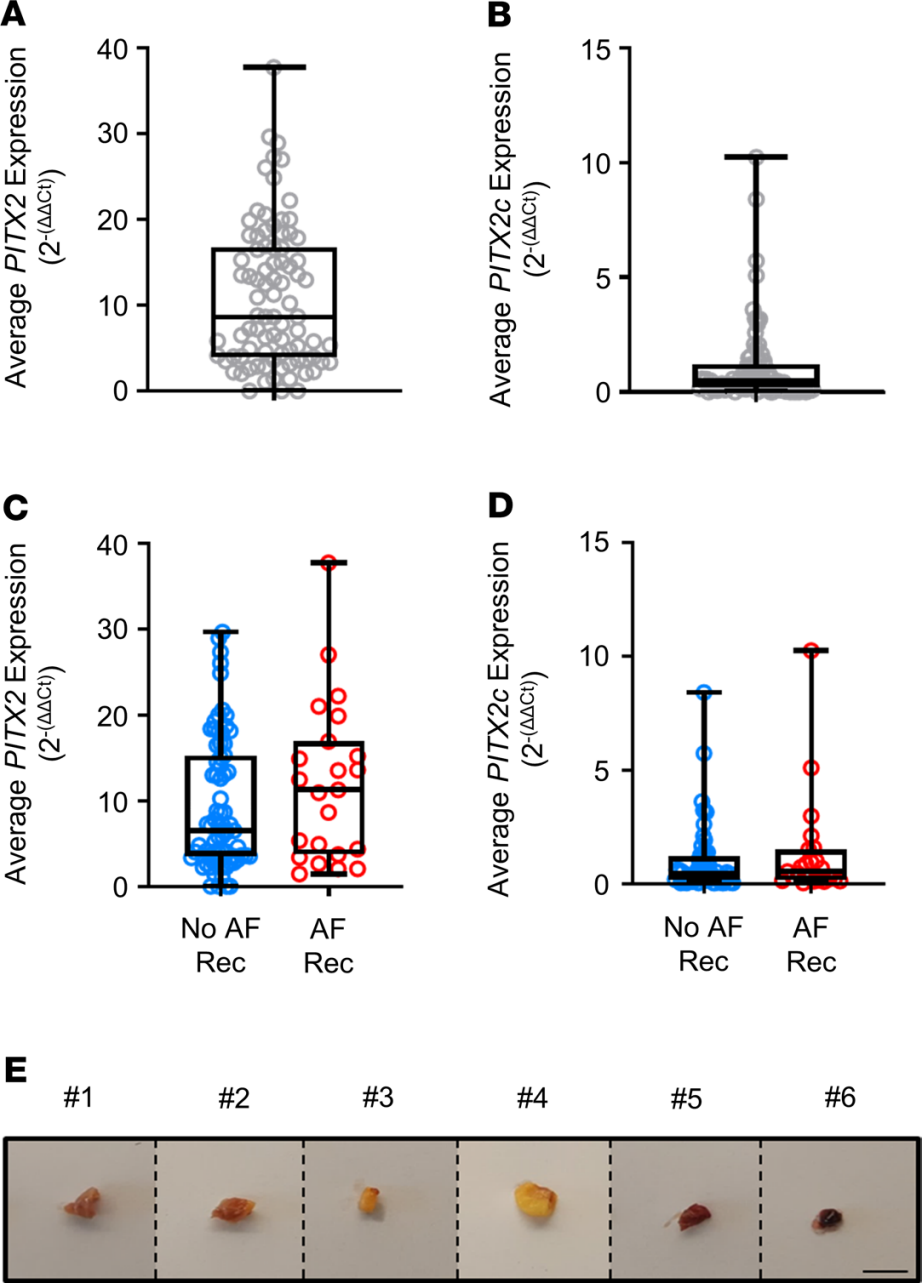
Expression of *PITX2* or *PITX2c* in whole left atrial tissue does not predict recurrent atrial fibrillation. Left atrial appendage samples were digested and assessed for levels of *PITX2* (**A**; *PITX2* median [Q1, Q3] 8.67 [3.90, 16.78]) and *PITX2c* (**B**; 0.47 [0.16, 1.20]) using qPCR. Results are expressed as an average normalized to 2 housekeeping genes (*GAPDH* and *POLR2A*) (*n* = 94). Expression levels of *PITX2* (**C**; *PITX2* No AF Rec 7.81 [3.96, 16.72]), Rec AF 11.28 [3.70, 16.96]; *P* = 0.704) and *PITX2c* (**D**; *PITX2c* No AF Rec 0.44 [0.18, 1.19], Rec AF 0.53 [0.16, 1.50]; *P* = 0.543) were stratified by clinical outcomes of having recurrent AF within 1 year after ablation surgery. AF Rec, patients with recurrent AF, *n* = 23; No AF Rec, patients without recurrent AF, *n* = 71. (**E**) Example biopsies of left atrial appendage tissue, highlighting tissue heterogeneity. Scale bar: 10 mm.

*PITX2* and *PITX2c* mRNA concentrations were quantified in 83 whole left atrial appendage tissue samples (Figure 1A). *PITX2* and *PITX2c* showed a widely variable distribution in expression in left atrial appendage whole tissue samples (Figure 2, A and B). *PITX2* concentrations were similar in patients with (median [Q1, Q3] 11.28 [3.70, 16.96]) and without AF recurrence (7.81 [3.96, 16.72], *P* = 0.704; Figure 2C). *PITX2c* concentrations also did not differ in patients with (0.53 [0.16, 1.50]) and without AF recurrence (0.44 [0.18, 1.19], *P* = 0.543; Figure 2D). Left atrial appendage whole tissue *PITX2* and *PITX2c* expression levels did not contribute to prediction of AF when considered with clinical characteristics. Morphological analysis of patient left atrial appendage tissue biopsies revealed tissue heterogeneity with marked fatty deposits and fibrosis in some specimens, and high myocardium content in others (Figure 2E).

### Left atrial cardiomyocytes are the main source of PITX2 in patients.

**Figure 3:**
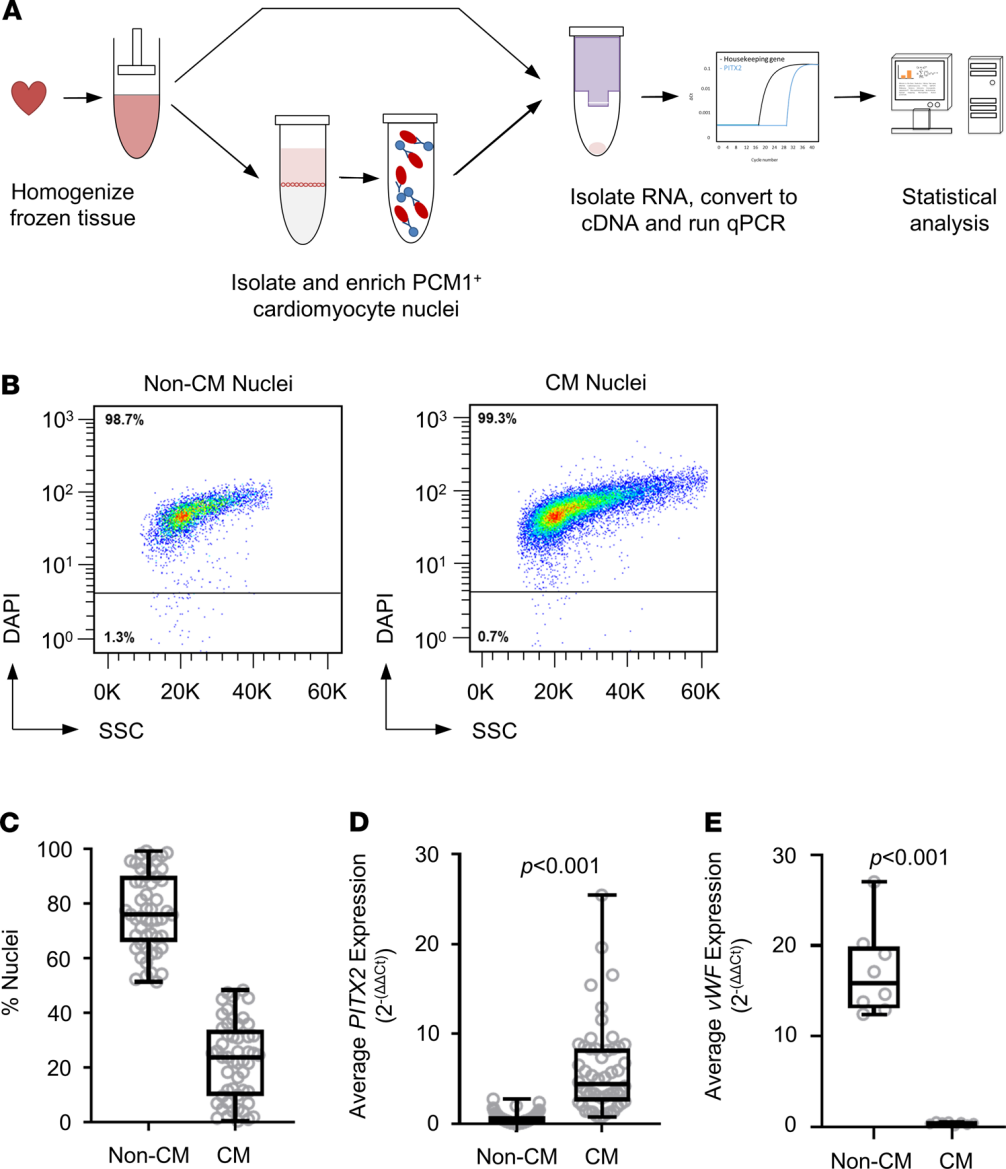
Assessing PITX2 levels in patient left atrial cardiomyocytes. (**A**) Overview of tissue processing and *PITX2* gene expression and analysis protocol. Nuclei were isolated from patient left atrial appendage samples, and cardiomyocytes (CM) were enriched using an anti-PCM1 antibody. Both PCM1-enriched (CM Nuclei) and -depleted (Non-CM Nuclei) fractions were harvested. (**B**) Quantity of nuclei was assessed by staining using DAPI and determined by flow cytometry. (**C**) The percentage of nuclei in either non-CM or CM fractions was calculated (Non-CM 76.09 [66.06, 90.22], CM 23.91 [9.78, 33.94]; *n* = 52). (**D**) Levels of *PITX2* (Non-CM 0.48 [0.19, 0.85], CM 4.43 [2.49, 8.39]; *P* < 0.001; *n* = 52) and (**E**) *vWF* (Non-CM 15.88 [13.12, 19.92], CM 0.44 [0.28, 0.58]; *P* < 0.001; *n* = 8) in both Non-CM and CM fractions were measured using qPCR. The results are expressed as an average normalized to 2 housekeeping genes (*GAPDH* and *POLR2A*). Statistical significance was calculated by using Mann-Whitney *U* test.

To assess the role of left atrial cardiomyocyte *PITX2* in recurrent AF, we quantified *PITX2* mRNA in cardiomyocyte and non-cardiomyocyte nuclei from another set of 52 left atrial appendage samples using a pericentriolar material–1 (PCM1) cardiomyocyte enrichment protocol (Figure 3A) (20). Cardiomyocyte quantity was assessed by DAPI staining and flow cytometry (Figure 3B). Approximately one-quarter of all nuclei were PCM1 positive (i.e., cardiomyocyte nuclei; Figure 3C) with marked variability (range 10%–60%), in line with the macroscopic appearance (Figure 2E). *PITX2* expression was largely confined to cardiomyocytes, and very low levels were detected in non-cardiomyocyte nuclei (0.48 [0.19, 0.85]; Figure 3D) in comparison with cardiomyocyte *PITX2* expression (4.43 [2.49, 8.39], *P* < 0.001; Figure 3D). Furthermore, expression of the endothelium-specific marker vWF was only detected in non-cardiomyocyte nuclei (15.88 [13.12, 19.92], cardiomyocyte 0.44 [0.28, 0.58], *P* < 0.001; Figure 3E), confirming the quality of the PCM1 enrichment preparations.

### Low left atrial cardiomyocyte PITX2 predicts recurrent AF after thoracoscopic AF ablation.

**Figure 4:**
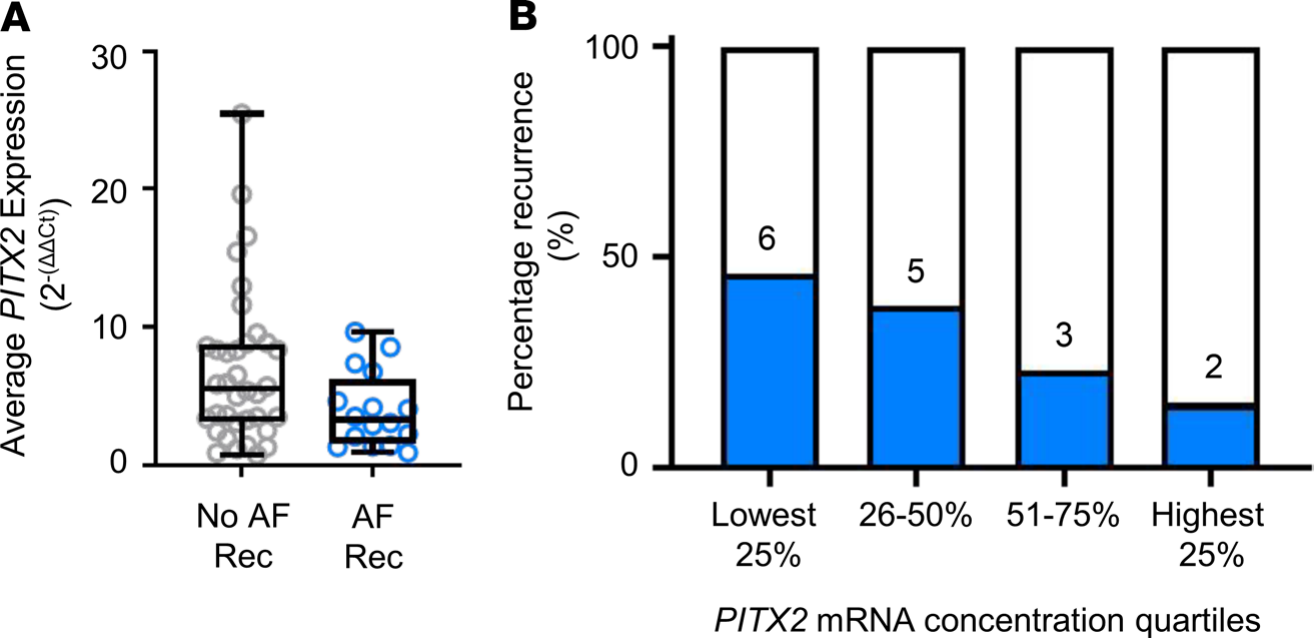
Reduced expression of *PITX2* in patient left atrial cardiomyocytes predicts recurrent atrial fibrillation. (**A**) Expression levels of *PITX2* in patient nuclei. Samples were stratified by AF recurrence at 1 year follow-up after ablation (No AF Rec 5.58 [3.16, 8.80], AF Rec 3.32 [1.60, 6.25]; *P* < 0.082. AF Rec, patients with recurrent AF within 1 year after ablation, *n* = 16; No AF Rec, patients without recurrent AF within 1 year after ablation, *n* = 36. (**B**) Stratification of *PITX2* mRNA concentrations from **A** into quartiles. The numbers of patients who experienced recurrent AF in the respective quartiles are shown.

Although the number of samples was limited, left atrial appendage cardiomyocyte *PITX2* concentrations were lower in patients with recurrent AF compared with patients without recurrence (Figure 4A, *P* = 0.082; Table 1). Multivariate analysis considering 4 clinical parameters shown to predict recurrent AF after ablation (21) and *PITX2* concentration with a forward selection process selected *PITX2* as the variable most strongly associated with AF recurrence (OR 0.840, 95% CI 0.695, 1.014), whereby every 2^–(ΔΔCt)^ increase in *PITX2* expression levels reduced the odds of recurrent AF by 16%. Although the confidence intervals encompassed the unity value of 1, the Hosmer-Lemeshow goodness-of-fit test indicated that the model was an adequate fit (*P* = 0.685). Stratification of *PITX2* mRNA concentrations into quartiles revealed that the frequency of recurrent AF increased with decreasing *PITX2* concentration (Figure 4B). While these analyses support the hypothesis that reduced left atrial cardiomyocyte *PITX2* concentrations are associated with recurrent AF in the first year after thoracoscopic AF ablation, they call for independent validation in a less-selected group of patients.

### Bmp10 is increased in murine left atria with reduced Pitx2c.

**Figure 5:**
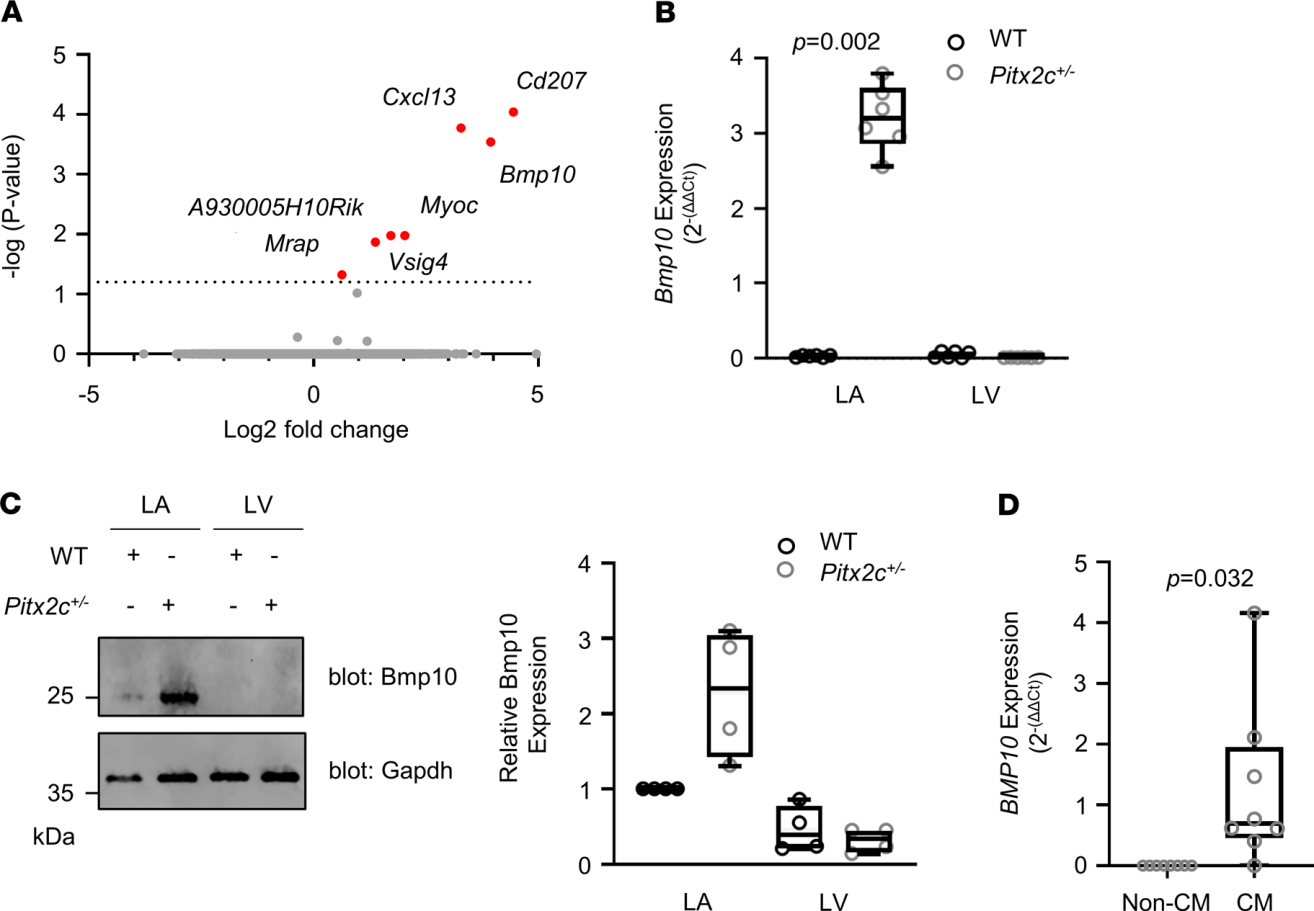
Bmp10 expression is increased following reduction of Pitx2. (**A**) RNA-Seq analysis of significantly upregulated genes in left atrial tissue from *Pitx2c^+/–^* mice (*n* = 3 mouse pairs). (**B**) *Bmp10* mRNA expression levels in the left atrium (LA) and left ventricle (LV) of WT and *Pitx2c^+/–^* mice, assessed by qPCR using *Gapdh* as a housekeeping gene (WT LA 0.03 [0.01, 0.04], *Pitx2c^+/–^* LA 3.20 [2.86, 3.60], *P* = 0.002; WT LV 0.05 [0.01, 0.09], *Pitx2c^+/–^* 0.01 [0.01, 0.02], *P* = 0.060; *n* = 6). Statistical significance was calculated using Mann-Whitney *U* test. (**C**) Protein expression of Bmp10 in the left atrium and left ventricle of WT and *Pixt2^+/–^* mice as assessed by Western blotting using Gapdh as a loading control (WT LA 1.00 [1.00, 1.00], *Pitx2c^+/–^* LA 2.34 [1.43, 3.05], *P* < 0.059; WT LV 0.40 [0.22, 0.78], *Pitx2c^+/–^* 0.34 [0.16, 0.45], *P* < 0.462; *n* = 4). (**D**) *BMP10* mRNA expression levels in human left atrial cardiomyocyte (CM) and non-cardiomyocyte (Non-CM) appendage samples assessed by qPCR using *GAPDH* as a housekeeping gene (Non-CM 0.00 [0.00, 0.00] CM 0.70 [0.45, 1.95]; *P* = 0.032, *n* = 8). Statistical significance was calculated using a 1-sample *t* test.

To identify heart-restricted, secreted proteins modulated by *PITX2*, we carried out unbiased RNA-Seq using left atria from WT and *Pitx2c^+/–^* mice (*n* = 3 paired mice; Figure 5A). This revealed *Cd207*, *Bmp10*, *Cxcl13*, *Myoc*, *Vsig4*, *A930005H10Rik*, and *Mrap* as the top 7 genes with differentially increased expression in left atrium of *Pitx2c^+/–^* mice. *Bmp10* was selected for further quantification due to its restriction to cardiac tissue and based on its known biology as a secreted protein (22). *Bmp10* mRNA, quantified by qPCR, was expressed at 32-fold-increased levels in the left atria of *Pitx2c^+/–^* mice compared with their WT littermates (WT 0.03 [0.01, 0.04], *Pitx2c^+/–^* 3.20 [2.86, 3.60], *P* = 0.002; Figure 5B) and at low to undetectable levels in left ventricular tissue of either genotype (WT 0.05 [0.01, 0.09], *Pitx2c^+/–^* 0.01 [0.01, 0.02], *P* = 0.060; Figure 5B). This result is consistent with prior reports (11, 12). Accordingly, Bmp10 protein concentrations were increased in the left atria of *Pitx2c^+/–^* mice (WT 1.00 [1.00, 1.00], *Pitx2c^+/–^* 2.34 [1.43, 3.05], *P* = 0.059; Figure 5C), while there was no change in Bmp10 protein concentrations in left ventricles (WT 0.40 [0.22, 0.78], *Pitx2c^+/–^* 0.34 [0.16, 0.45], *P* = 0.462; Figure 5C; see Supplemental Figure 1 for full Western blot gel; supplemental material available online with this article; https://doi.org/10.1172/jci.insight.139179DS1). *BMP10* was mainly expressed in cardiomyocytes obtained from human left atrial appendages (non-cardiomyocytes 0.00 [0.00, 0.00], cardiomyocytes 0.70 [0.45, 1.95], *P* = 0.032; Figure 5D). These findings suggest that *BMP10* is repressed by *PITX2* in the adult left atrium. Importantly, unlike markers such as N-terminal pro–B-type natriuretic peptide (NTproBNP), plasma concentrations of BMP10 appear relatively unaffected by other cardiovascular conditions such as heart failure (22). Hence, elevated plasma BMP10 concentrations were used as a surrogate for reduced atrial *PITX2*.

### Elevated blood BMP10 protein concentrations are associated with recurrent AF after AF ablation.

**Figure 6:**
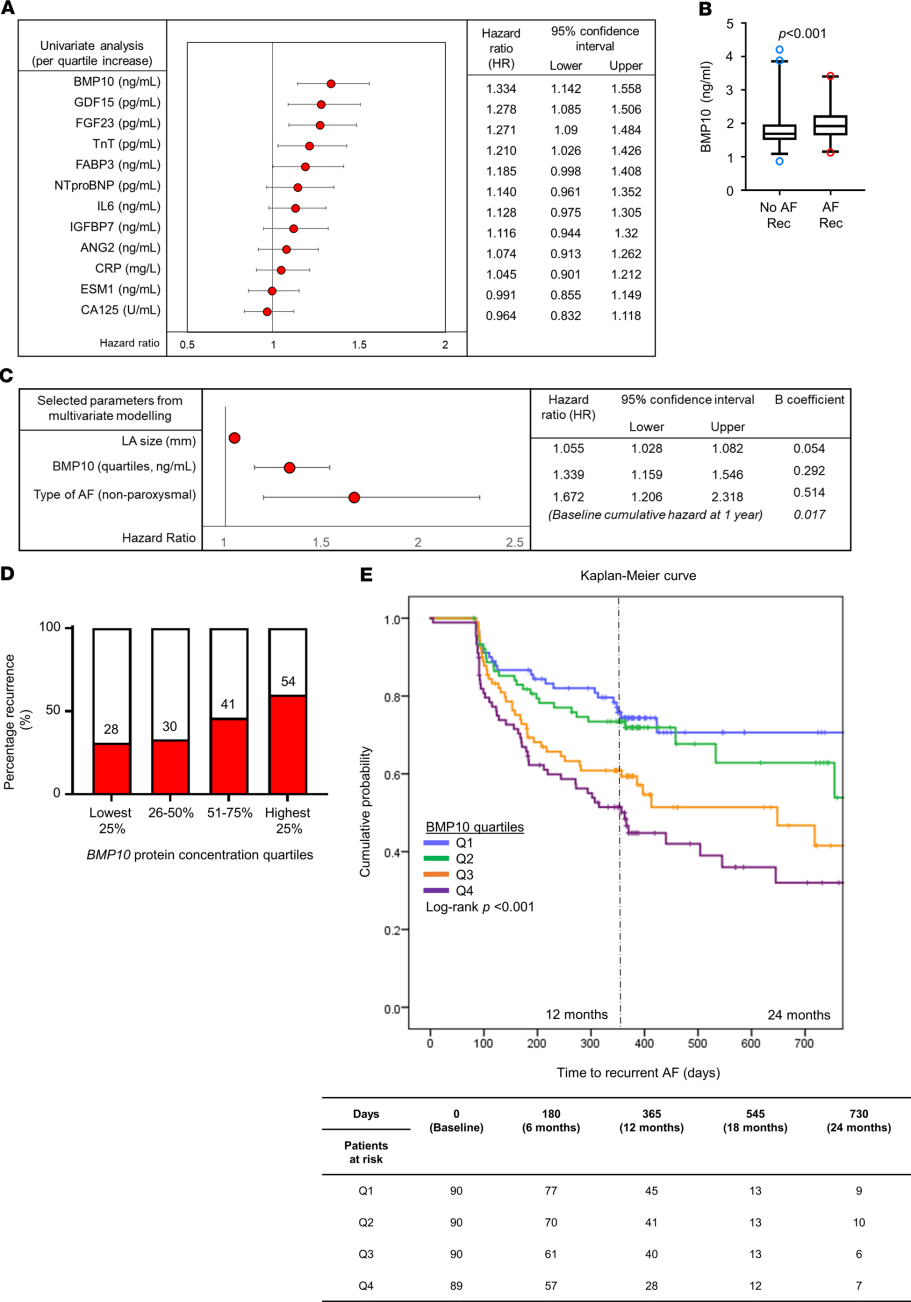
Increased BMP10 predicts recurrent atrial fibrillation in 359 patients after catheter ablation. (**A**) By univariate analysis, BMP10 confers the highest relative risk for recurrent AF among 11 other common cardiovascular biomarkers after adjustment for age, sex, type of AF, and left atrial diameter. (**B**) BMP10 levels are significantly elevated in patients with recurrent AF. (**C**) In multivariate analysis, increased left atrial (LA) size, nonparoxysmal type of AF, and elevated BMP10 predict recurrent AF. (**D**) When patients were stratified into quartiles based on BMP10 concentration, the largest proportion of patients with recurrent AF were in the highest BMP10 quartile. The numbers of patients that experienced recurrent AF in the respective quartiles are shown. (**E**) Stratification of patients by BMP10 quartiles corresponds with their rhythm outcome up to 2 years follow-up, with the worst outcomes in patients in the highest quartile (Q4). ANG2, angiopoietin 2; CRP, high-sensitivity C-reactive protein; CA125, cancer antigen 125; ESM1, endothelial cell–specific molecule 1; FABP3, fatty acid binding protein 3; GDF15, growth differentiation factor 15; IGFBP7, insulin like growth factor binding protein 7; TnT, high-sensitivity cardiac troponin T.

In the AFLMU cohort (see Methods; Figure 1B and Table 2), patients with and without recurrences did not significantly differ in terms of hypertension, heart failure, diabetes, stroke/transient ischemic attack (TIA), or BMI status. In univariate analysis adjusted for age, sex, type of AF, and left atrial diameter, BMP10 conferred the highest relative risk among 12 tested biomarkers (HR per quartile increase 1.334, 95% CI 1.142, 1.558; Figure 6A). Patients with recurrent AF had significantly higher BMP10 levels (1.93 [1.66, 2.26], *n* = 153) compared with patients without recurrent AF (1.68 [1.51, 1.97], *P* < 0.001, *n* = 206; Figure 6B).

BMP10 was then combined with 4 established clinical characteristics predictive of recurrent AF (age, sex, AF pattern, left atrial diameter) in a Cox regression with forward selection (entry criterion, *P* = 0.05) to determine the most parsimonious multivariate model. The best combination of variables for achieving a significant prediction for recurrent AF consisted of (in order of entry) BMP10, left atrial size, and type of AF (Figure 6C). This model had an area under the ROC curve (AUC) of 0.689 [0.633, 0.744]. To adjust for overoptimism, the model was bootstrapped (1000 samples), with very little bias detected (Supplemental Figure 2). We also considered all 12 biomarkers in the model with forward selection. The best combination of variables remained the same as above — BMP10, left atrial size, and type of AF, with the addition of FGF23 (Table 3). The addition of FGF23 marginally improved the performance of the model (AUC 0.693 [0.638, 0.748]). Sensitivity analyses using LASSO for data reduction yielded the same predictors as forward selection in all instances (see Supplemental Methods, “LASSO for data reduction,” and Supplemental Table 1). To reduce variability in the Cox regression modeling secondary to the range of follow-up durations (median [Q1, Q3] 358 [173, 392] days), we included a sensitivity analysis using logistic regression, which removes the time component of the model (Supplemental Methods, “Logistic regression”). The sensitivity analysis results showed trends nearly identical to those observed in our main analysis.

When patients were stratified into quartiles by BMP concentrations, the highest quartile had the largest proportion of patients with recurrent AF (χ^2^ *P* < 0.001, Figure 6D; see Supplemental Figure 2 for other cardiovascular biomarkers) and the lowest survival probability compared with other quartiles (log-rank *P* < 0.001; Figure 6E). Thus, increased BMP10 blood levels confer the highest relative risk of recurrent AF, both univariately (adjusted and unadjusted) and in the presence of other well-known cardiovascular biomarkers and established clinical predictors.

## Discussion

### Main findings.

Low left atrial cardiomyocyte *PITX2* concentrations appear to be associated with an increased risk of recurrent AF after thoracoscopic AF ablation. Furthermore, elevated blood BMP10 protein concentrations, a new biomarker for AF quantifying a secreted, *PITX2*-controlled left atrial protein, predict recurrent AF after catheter-based AF ablation in patients. These results can inform future strategies to prevent recurrent AF in patients, e.g., targeting those with low left atrial *PITX2* or high blood BMP10 levels.

**Figure 7:**
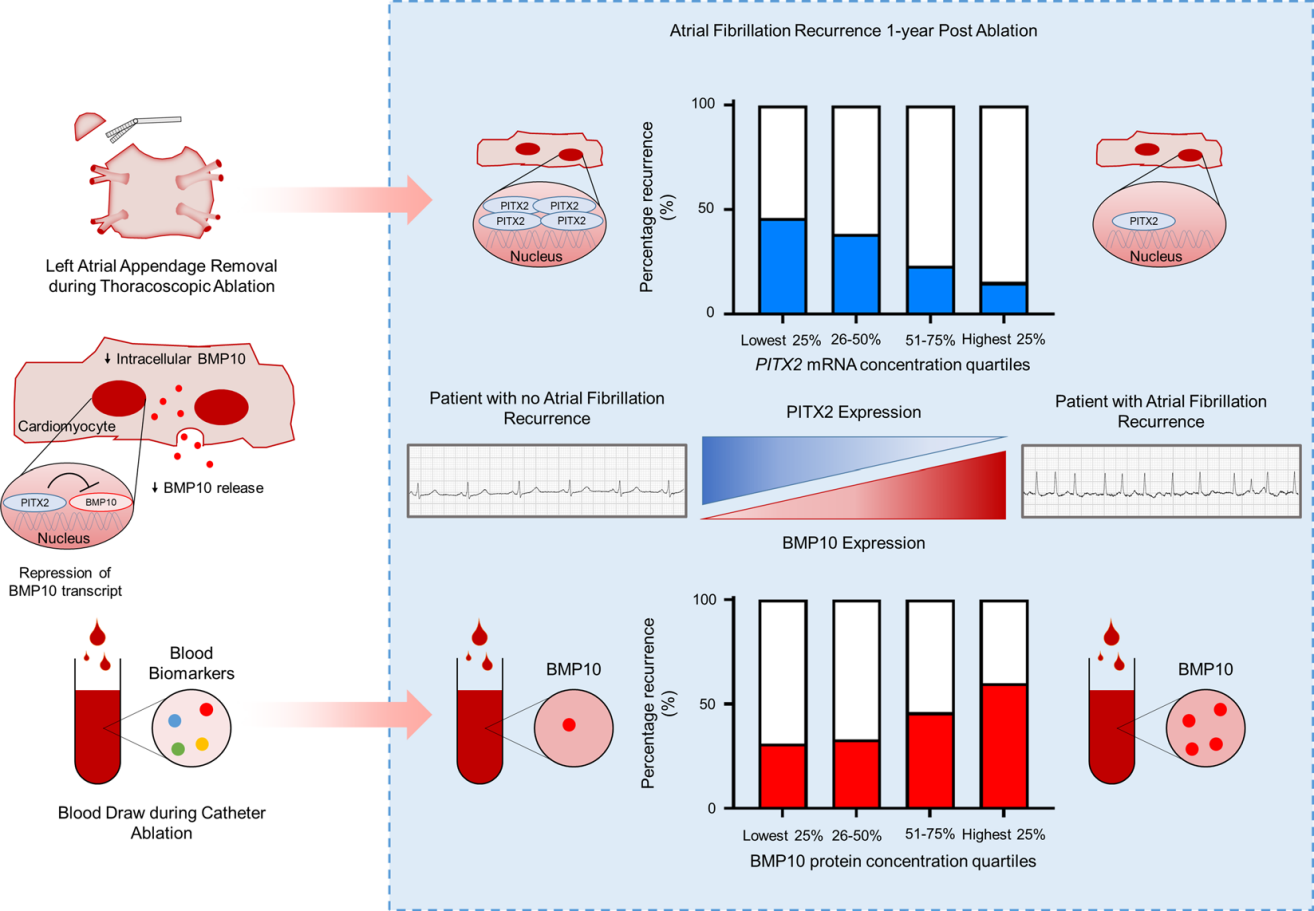
Correlation of low left atrial cardiomyocyte *PITX2* mRNA and elevated BMP10 protein concentrations with recurrent AF after ablation. Our data show that left atrial cardiomyocyte *PITX2* mRNA concentrations, measured in left atrial appendages excised after thoracoscopic AF ablation, are a strong predictor of recurrent AF after AF ablation. Based on molecular biology analyses, we postulate that PITX2 represses production of the left atrial protein BMP10 that is secreted into blood. Indeed, elevated concentrations of BMP10 in peripheral blood were found to predict recurrent AF after AF ablation. These data call for validation in independent cohorts.

Although many patients with AF respond to rhythm control therapy, others experience early recurrences: symptomatic recurrence of AF occurs within 6–12 months in 40%–70% of patients on antiarrhythmic drug therapy (23–25) and in 20%–50% after AF ablation (AFLMU cohort in this study and refs. 26–28). Current practice leaves selection of rhythm control therapy to local protocols (29). The reasons for recurrent AF after ablation are not fully understood (30, 31), although there is clinical evidence that common gene variants on chromosome 4q25, close to the *PITX2* gene, are associated with recurrent AF after ablation (3, 5, 32). This study identifies reduced left atrial cardiomyocyte *PITX2* concentrations and its surrogate, elevated BMP10 concentrations, as a major predictor of recurrent AF after ablation (Figure 7). The effect of low left atrial *PITX2* on recurrent AF was found only in left atrial cardiomyocyte preparations, but not in whole left atrial tissue, consistent with previous findings in whole left atrial tissue from patients (15, 33).

*PITX2* functions as an essential cardiac transcriptional factor, possibly acting within a network of transcriptional regulation (18). Reduced *PITX2* expression results in congenital heart diseases (12, 34) and cardiac arrhythmogenic defects (14). While a direct link between AF and *PITX2* has so far only been shown in murine models, single nucleotide polymorphisms at the 4q25 locus (the strongest genomic markers of AF risk) can regulate *PITX2* expression (35) and subsequently alter its transcriptional activity. Reduced left atrial *PITX2* can modify ion channels and cell-cell contacts, thus changing their electrical function, resulting in a predisposition to AF in mice (12–14, 16, 17). Our results provide the first evidence to our knowledge that low left atrial cardiomyocyte *PITX2* levels contribute to recurrent AF after ablation in patients in whom left atrial tissue was collected at the time of AF ablation.

Our results also confirm recent data from mouse atria indicating that cardiomyocytes are the major cell type expressing *PITX2* in the adult left atrium (36). Furthermore, we found that approximately one-quarter of nuclei in human left atrium are cardiomyocyte, consistent with prior data in mice (36). The marked variability in cardiomyocyte content of the left atrium, dependent, e.g., on the degree of atrial fibrosis and atrial fatty infiltration (Figure 2E), can explain why *PITX2* concentrations in whole atrial tissue were not associated with recurrent AF.

Our gene expression analyses identified *Bmp10* as a gene whose expression is increased when *Pitx2c* is reduced, consistent with *PITX2* repressing BMP10. These findings are consistent with other murine models of *Pitx2* deficiency (10–12). In addition, recent findings in a mouse model that deactivated the enhancer region of *Pitx2c* found *Bmp10* to be one of the most upregulated genes (37). These results suggest that a common repressor/enhancer transcriptional network may exist between *Bmp10* and *Pitx2* whereby the loss of one of these genes results in the reciprocal upregulation of the other (18, 38). Such findings are plausible given that *PITX2* is known to be a key regulator of “leftness” in the heart during development (36, 39) and *BMP10* is a right atrial gene (11, 40). Additional features supported our selection of increased BMP10 plasma concentrations as a surrogate marker for reduced left atrial *PITX2*. BMP10 is a secreted protein that is released into plasma (41). BMP10 is a heart-restricted protein, expressed in cardiomyocytes during development and required for cardiomyocyte growth and development (42, 43) and with little expression in the left ventricle (Figure 5, B and C).

Using BMP10 as a plasma surrogate for left atrial *PITX2*, we found that elevated BMP10 concentrations, quantified just before a clinically indicated AF ablation procedure, are a good predictor of recurrent AF after ablation (Figure 6). BMP10 improved prediction of recurrent AF when added to established clinical features that predict recurrent AF (21). The predictive power of BMP10 outperformed other plasma biomarkers that have been proposed as predictors of recurrent AF (44–47).

Taken together, our findings provide further support for the hypothesis that reduced left atrial cardiomyocyte *PITX2* contributes to recurrent AF. These results can inform strategies to prevent recurrent AF in patients, e.g., targeting those with low levels of *PITX2*.

### Strengths.

First, left atrial *PITX2* was quantified in patients undergoing stand-alone thoracoscopic AF ablation, rather than patients requiring open heart surgery, who are the source of most analyses of human left atrial tissue. This population comprised a small subset of patients with AF receiving rhythm control therapy, enriched for patients with recurrent AF after AF ablation, rather than being a population of patients with several other cardiovascular diseases requiring surgery. This is a strength, as patients undergoing thoracoscopic AF ablation are more similar to patients receiving rhythm control therapy for AF than patients undergoing open heart surgery for other conditions, who are a common source for atrial tissue, but also calls for validation in additional patient cohorts treated with rhythm control therapy.

Until the present study, *PITX2* expression had largely been investigated in whole tissue left atrial appendage samples, rendering measured concentrations subject to interference by non-cardiomyocyte fractions (Figure 2E and refs. 15, 33, 48). The cardiomyocyte isolation protocol applied here enriches the nuclear fraction of cardiomyocytes and allows for a purer analysis of nuclear cardiomyocyte genes.

### Limitations.

First, the AFACT and MARK AF cohorts used in the statistical analysis are large for a study involving thoracoscopically collected left atrial tissue in patients, but relatively small for a clinical study employing multivariate analysis. Although, this limits the power to detect additional factors associated with recurrent AF, it was not possible to obtain more tissue samples. Therefore, we validated our findings in an independent cohort by studying a secreted form of BMP10, which was identified as a *PITX2*-regulated gene. Second, while *BMP10* was identified by an atrial gene expression screen using established models for reduced *PITX2* expression, which is in agreement with published data, further experiments, i.e., ChIP-Seq or assay for transposase-accessible chromatin using sequencing (ATAC-Seq), are warranted to demonstrate directly that *BMP10* is controlled by *PITX2*. Third, while the AFLMU data set is rather large for an AF ablation cohort with biomarkers, further studies in independent patient data sets, ideally assessing atrial *PITX2* and plasma BMP10 concentrations in the same patients, are warranted to confirm our findings. Further exploratory analyses, potentially including machine learning approaches in addition to established methodologies, can shed further light on the complex regulation of left atrial gene expression. Finally, this study did not address whether common gene variants on chromosome 4q25 regulate *PITX2*. While cellular expression of such variants can reduce (35) or increase *PITX2* levels (49), the control of *PITX2* in the left atrium of patients will be modified by transcriptional and epigenetic regulation of *PITX2*, along with other factors (reviewed in ref. 18). The partial redundancy in the regulation of atrial gene expression (18) can further mitigate the AF drivers associated with reduced *PITX2*.

### Conclusions.

Low left atrial cardiomyocyte *PITX2* and elevated blood BMP10 predict recurrent AF after catheter-based AF ablation in patients. BMP10 emerges as a promising plasma biomarker to assess left atrial *PITX2* activity. These results can inform future strategies to prevent recurrent AF in patients, e.g., targeting those with low *PITX2*.

## Methods

### Study populations

#### AFACT.

Atrial Fibrillation Ablation and Autonomic Modulation Via Thorascopic Surgery (AFACT; NCT01091389) is a prospective, randomized, controlled, single-center study (Amsterdam UMC) that recruited participants between April 2010 and January 2015 to investigate the efficacy and safety of ganglion plexus ablation in patients undergoing thoracoscopic AF ablation. Details of inclusion and exclusion criteria as well as the main outcomes of this study have been previously published (50).

#### MARK AF.

The MARK AF study (ethical approval NL50069.018.14) recruited consecutive patients undergoing thoracoscopic AF ablation. It was designed as a prospective registry collecting data from patients not included into AFACT but seen for thoracoscopic AF ablation at Amsterdam UMC. Patients with AF were recruited using the same criteria as for the AFACT study (50).

#### AFLMU.

The AFLMU study (EK494-16) is an ongoing prospective research project. For this analysis, only patients enrolled until 2016 were considered to enable meaningful follow-up (Figure 1B). For this analysis, patients undergoing AF ablation were considered. Blood samples were collected during the ablation from a groin puncture site and before access to the left atrium.

Patients underwent systematic rhythm follow-up with 24-hour Holter monitoring every 3 months (AFACT and MARK AF) or 7-day Holter monitoring (AFLMU; Figure 1). All patients were of European ancestry. ECG-documented AF recurrences were reviewed by an experienced operator before counting.

### Biological samples

#### Left atrial and left ventricular tissue preparation.

Left atrial appendages were collected from patients in the AFACT and MARK AF studies during thoracoscopic AF ablation and frozen at –80°C for later analysis. Murine left atrial and left ventricle tissue were harvested from 10 pairs of 2- to 3-month-old WT and *Pitx2c^+/–^* mice bred on a MF1 genetic background and snap-frozen in liquid nitrogen. The *Pitx2c^+/–^* mice were originally obtained from Nigel Brown (St George’s University, London, United Kingdom) and have previously been characterized (10). All molecular biology experiments performed by investigators blinded to rhythm outcome or mouse genotype (see Supplemental Methods for details).

#### RNA-Seq and molecular biology in left atrial mouse tissue.

Whole tissue left atrium samples from 6 pairs of 3-month-old WT and *Pitx2c^+/–^* mice were snap-frozen in liquid nitrogen and stored at –80°C.

#### Blood biomarkers.

BMP10 levels were quantified in ng/mL from EDTA plasma using a pre-commercial high-throughput assay on a cobas Elecsys platform (Roche Diagnostics) employing Elecsys Electro-ChemiLuminescence (ECL) technology. By calibrating with serial dilutions of recombinant BMP10, the instrument read-out was precisely normalized across runs to enable large cohort measurements with a high degree of accuracy. A total of 11 cardiovascular biomarkers that have been proposed as predictors of AF were quantified as well (angiopoietin 2; high-sensitivity C-reactive protein; cancer antigen 125; endothelial cell–specific molecule 1; FGF23; fatty acid binding protein 3; growth differentiation factor 15; insulin-like growth factor binding protein 7; IL-6; NTproBNP; high-sensitivity cardiac troponin T). All measurements were done by investigators blinded to clinical information and outcomes.

See Supplemental Methods for further technical details.

### Data analysis

#### RNA-Seq analysis.

RNA-Seq FASTQ files were aligned on HISAT2 (version 2.1.0) using Ensembl *Mus musculus* reference GRCm38.91 (51, 52). Aligned reads were counted using HTSeq version 0.9.1 (53). Required transformations through different RNA-Seq analysis steps were done using SAMtools version 1.4 (http://samtools.sourceforge.net/). Differential expression was obtained using DESeq2 in R. Ensembl IDs were transformed to gene symbols using BioTools (https://www.biotools.fr/) Data were deposited in the NCBI’s Gene Expression Omnibus database (GEO GSE152181).

#### Statistics.

For molecular biology experiments, Mann-Whitney *U* test was used for all analyses looking at between-group comparisons and *t* test when direct comparisons were made to normalized data involving a single value. All data were tested for normality using a Shapiro-Wilk test. Box-and-whisker plots display the 1st–99th percentile, and *P* values are stated for statistically significant comparisons. For direct comparisons between 2 groups, median values along with Q1 and Q3 are stated in the text and figure legends. Biomarkers were tested for association with outcomes per quartile increase for comparability between biomarkers.

The baseline characteristics of patients with and without AF recurrence at 1 year follow-up in AFACT and MARK AF cohorts were compared. Only recurrences after 3 months after ablation were considered. Categorical variables were assessed using χ^2^ tests. Continuous variables were compared using independent-samples *t* tests or Mann-Whitney *U* tests as applicable after testing for normality using the Kolmogorov-Smirnov test. A 2-tailed *P* value less than 0.05 was considered to be statistically significant.

A logistic regression model was fitted with forward selection (entry criterion, *P* = 0.1) to identify parameters associated with increased odds of AF recurrence among the top 4 clinical predictors of recurrent AF after catheter ablation and quantified *PITX2/PITX2c* expression levels in whole atrial tissue or cardiomyocyte samples. Clinical predictors were identified from a systematic review (21). LASSO performed as sensitivity analyses as an alternative selection procedure. OR and 95% CIs were calculated for all selected variables.

A Cox regression model was applied using data from the AFLMU cohort, with *PITX2/PITX2c* being replaced by BMP10. BMP10 was also compared with 11 other cardiovascular biomarkers. All analyses were performed using SPSS v.24 (IBM Corp.). Authors had direct access to primary data from all the studies above for data analysis. All graphs were produced using GraphPad Prism8 software.

#### Study approval.

For human studies, all patients provided written informed consent, and studies were approved as follows: AFACT (NCT01091389) — The study conformed to the Declaration of Helsinki, and all patients provided written informed consent; MARK AF (NL50069.018.14) — All patients provided written informed consent, and the study was approved by the ethics committee of Amsterdam Medical Center; AFLMU — All patients provided written informed consent, and the study was approved by the Ethics Committee of LMU Munich (EK494-16). Experiments involving the use of murine tissue were performed under a protocol approved by the University of Birmingham Animal Welfare and Ethics Review Body guidelines (Home Office license PFDAAF77F).

## Author contributions

JSR designed, performed, and analyzed the experiments shown in Figures 2–5 and Figure 7; designed the study; and wrote the manuscript. WC designed, performed, and analyzed experiments shown in Figures 1, 6, and 7, Tables 1–3, Supplemental Figures 2–4, and Supplemental Tables 1–3; designed the study; and wrote the manuscript. VRC, AW, GVG, and MS performed and interpreted the RNA-Seq analyses. PMK performed biomarker quantification experiments. SNK prepared samples used for Figure 4. RW and JRDG provided AFACT and MARK AF samples and clinical data used in the study. MFS and SK provided AFLMU plasma samples and clinical data. APH and DP provided input into interpretation of the murine and biomarker data. LF and PK designed and coordinated the study and wrote the manuscript. All authors reviewed the results, contributed to the manuscript, and approved the final version of the manuscript.

## Supplementary Material

Supplemental data

ICMJE disclosure forms

## Figures and Tables

**Table 3:**
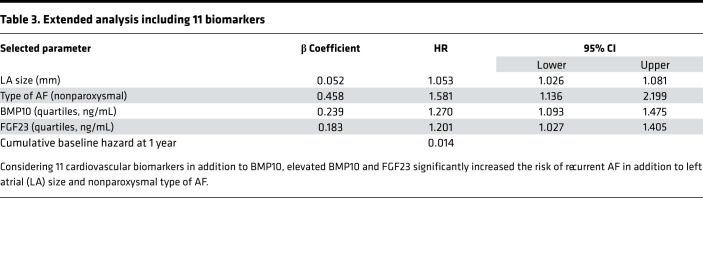
Extended analysis including 11 biomarkers

**Table 2:**
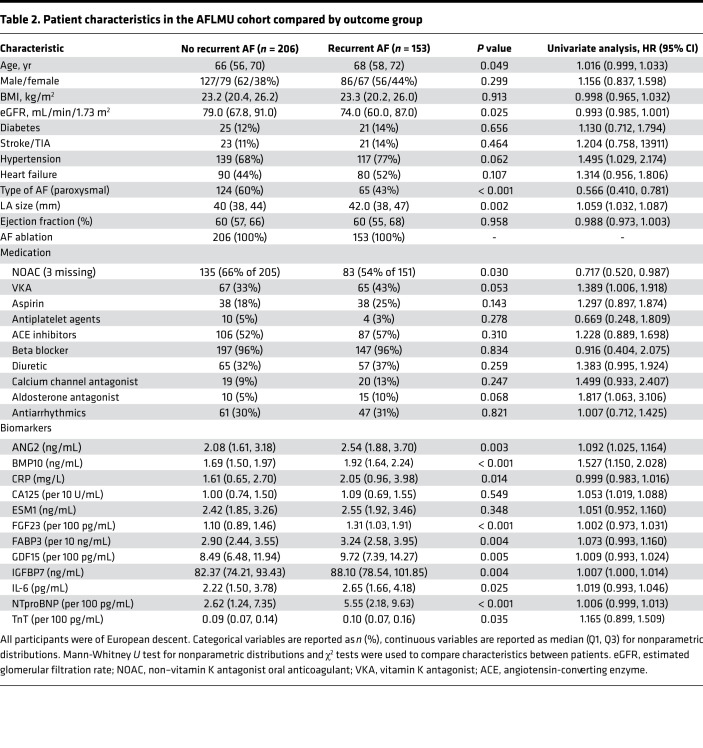
Patient characteristics in the AFLMU cohort compared by outcome group

**Table 1:**
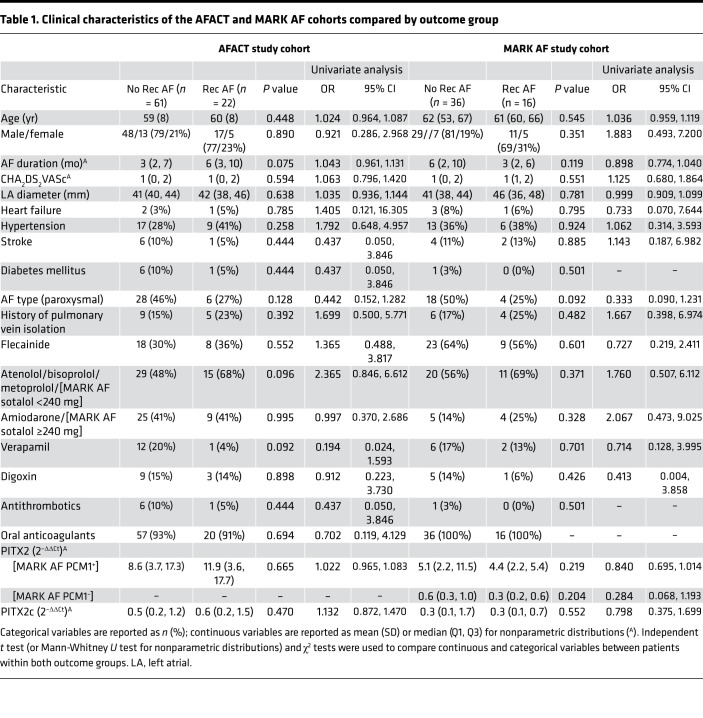
Clinical characteristics of the AFACT and MARK AF cohorts compared by outcome group

## Version 1 08/20/2020

Electronic publication
