# Supplementary material for: Reduced left atrial cardiomyocyte PITX2 and elevated circulating BMP10 predict atrial fibrillation after ablation
Source: JCI Insight. 2020 Aug 20;5(16):e139179. doi: 10.1172/jci.insight.139179 (PMC7455124; doi:10.1172/jci.insight.139179)
Supplement: Supplemental data [file jciinsight-5-139179-s238.pdf]

Reduced left atrial cardiomyocyte PITX2 and elevated circulating BMP10 predict atrial fibrillation after ablation

Jasmeet S. Reyat[1*], Winnie Chua[1*], Victor R. Cardoso[1,2], Anika Witten[3], Peter M Kastner[4], S. Nashitha Kabir[1], Moritz F. Sinner[5,6], Robin Wesselink[7], Andrew P. Holmes[1], Davor Pavlovic[1], Monika Stoll[3,8], Stefan Kääb[5,9], Georgios V. Gkoutos[1,2,10], Joris R. de Groot[7], Paulus Kirchhof[1,9,11,12], Larissa Fabritz[1,9,11]

Supplemental Material

Extended Methods

Tissue processing and RNA isolation. Left atrial appendage tissue samples were digested using a previously described protocol (1). Briefly, whole tissue samples (30-50mg) were homogenized in Buffer RLT (Qiagen, Germany) using a Precellys® 24 tissue homogenizer (Berlin Instruments, Germany). Whole tissue left atrial and left ventricle tissue samples from wild type (WT) or $Pitx2c^{+/-}$ were homogenized similarly. For isolation of cardiomyocytes, a pericentriolar material-1 (PCM1) enrichment protocol was utilized (2). Tissue (≥200mg) was initially homogenized in HB Buffer (0.25M sucrose, 25mM KCl, 5mM $MgCl_2$, 20mM Tricine-KOH pH 7.8) containing protease inhibitors (Roche, Switzerland), Spermine (Sigma-Aldrich, USA), Spermidine trihydrochloride (Sigma-Aldrich) and RNasin (Promega, USA). The tissue homogenate was then layered onto a gradient of OptiPREP (Sigma-Aldrich) and centrifuged at 10,000 x g. Nuclei were transferred into Eppendorf tubes containing Dynabeads (Sigma-Aldrich) conjugated to an anti-PCM1 antibody (HPA023370, Sigma-Aldrich) and incubated at 4°C. The samples were magnetically sorted into PCM1 enriched and depleted fractions. RNA was isolated from either whole tissue or nuclei using the RNeasy Mircro Kit (Qiagen, Germany) according to the manufacturer's instructions. RNA concentrations were measured with the NanoDrop ND-100 (Thermo Scientific, USA) and stored at -80°C until use.

Quantification of nuclei. A sample of the purified nuclei was used to quantitate the number and viability of the nuclei by flow cytometry. Briefly, nuclei samples were spun down at 300 x g and resuspended in FACS buffer (0.03% BSA and 0.01% NaN_3 in PBS) containing an antibody to DAPI (Thermo Scientific, USA). Samples were kept on ice for 30 minutes before being washed twice using FACS buffer and subsequently processed on a CyAn ADP flow

cytometer (Dako, the Netherlands). Data analysis was carried out using FlowJo software (FlowJo, USA).

<u>*cDNA conversion and qPCR.*</u> 1µg of isolated RNA was converted to cDNA using the High Capacity cDNA Reverse Transcription Kit (Applied Biosystems, USA) according to the manufacturer's instructions using standard cycling conditions. The reaction was run in a Sensquest Labcycler (Geneflow, UK) at 25°C for 10 minutes, 37°C for 120 minutes, 85°C for 5 minutes with a 4°C hold temperature. Synthesized cDNA was stored at -20°C until further use. For the qPCR reaction, a master mix solution consisting of 10µl TaqMan® Universal Primer MasterMix (Thermo Scientific), 1µl of primer (*PITX2* – Hs0434069_mH, *PITX2c* – Hs01553178_g1, *BMP10* – Hs00205566_m1, *vWF* – Hs01109446_m1, *GAPDH* – Hs99999905_m1, *POLR2A* – Hs00172187_m1, *Bmp10* – Ms01183889_m1, *Gapdh* – Mm99999915_g1) and 2µl of cDNA at a concentration of 5ng/ml was prepared. The qPCR was performed using the 7500 Real-Time PCR system (Thermo Scientific) that had been calibrated to detect FAM fluorescent probes using standard cycling parameters. For human samples, the absolute expression levels of genes relative to *GAPDH* and *POLR2A* were calculated using the ΔΔCt method (3). Where undetectable levels of gene expression were observed, the lowest detectable expression value was used. Similarly, for assessing gene expression in murine samples, the ΔΔCt method was applied using *GAPDH* as a housekeeping gene.

<u>*Murine tissue processing for RNA-Seq.*</u> Tissue samples were immediately snap-frozen for digestion and RNA extraction. Quality controlled total RNA with a RNA Integrity Number (RIN) >7 was measured using an Agilent Bioanalyzer. After quantification using a Qubit RNA Assay Kit (Life Technologies), RNA was enriched for the Poly(A) RNA fraction by using the NEBNext Poly(A) mRNA Magnetic Isolation Module (NEB). Subsequent Next Generation Sequencing cDNA library preparation was carried out using the NEBNext Ultra RNA Library Prep Kit for Illumina (NEB) according to the manufacturer's instructions. The size of the resulting library was checked (Agilent Bioanalyzer High Sensitivity DNA Analysis Kit) and quantified by qPCR (NEBNext Library quant Kit for Illumina, NEB). Equimolar pooled libraries were sequenced in single read mode (80 cycles) on a NextSeq500 machine (Ilumina) and v2 chemistry. De-multiplexing was performed using Bcl2Fastq v2.20.4.422 tool (Illumina) followed by adaptor trimming. In addition, a quality control check using Cutadapt was used.

<u>*Western blotting.*</u> Whole tissue left atrial and left ventricle tissue samples from WT or *Pitx2c*$^{+/-}$ were homogenized in 100µl homogenization buffer (1.2% Tris-HCL in dH_2O) containing protease and phosphatase inhibitors (Thermo Scientific, USA) using a Precellys® 24 tissue homogenizer (Berlin Instruments, Germany). Protein concentration was calculated

using a BCA assay (*DC* Protein Assay Kit, Bio-Rad, USA) and a total of 20µg of lysate was resolved on a 4-20% Tris-Glycine SDS-Page gel (Bio-Rad, USA). The gel was transferred using a TransBlot® Turbo^{TM} Mini PVDF Transfer Pack (Bio-Rad, USA) and the membrane was blocked in Odyssey Blocking Buffer (LI-COR, UK). The membrane was incubated overnight at 4°C with Mouse anti-BMP10 (#462732, R&D systems, UK) and Rabbit anti-GAPDH (14C10, Cell Signaling, The Netherlands) antibodies diluted in 3% BSA in TBST. The membrane was then incubated with fluorescent Mouse800 and Rabbit680 LI-COR secondary antibodies (LI-COR, UK) before being imaged using the LI-COR Fc Dual-Mode Imaging System (LI-COR, UK). Protein expression was quantified using the LI-COR Image Studio Lite Software (LI-COR, UK) and reported as relative expression to GAPDH.

Biomarker quantification. Absolute protein concentrations were centrally quantified (Roche Diagnostics, Penzberg, Germany) in EDTA plasma. Seven proteins, CA125, Growth Differentiation Factor-15 (GDF-15), Interleukin-6 (IL-6), N-terminal pro B-type natriuretic peptide (NT-proBNP), cardiac Troponin T (cTnT), Creatinine and Cardiac C-Reactive Protein (CRP), were measured with commercialised commercially available Roche Eleimmunoassays analysers (in-vitro diagnostic tests (test names) on a cobas Elecsys® e 601 analyser (for CA125 II, GDF-15, IL-6, NT-proBNP II, Troponin T hs) and ; cobas c 501 for Crea-E (creatinine and, CRPHS; Roche Diagnostics, Mannheim, Germany). A further six proteins, Angiopoietin-2 (ANG2), Bone Morphogenetic Protein 10 (BMP10), Endothelial Specific Molecule 1 (ESM-1), Heart Fatty Acid Binding Protein (FABP-3), Fibroblast Growth Factor 23 (FGF23), and Insulin-like Growth Factor-binding Protein 7 (IGFBP7) were quantified using developed pre-commercial high-throughput Elecsys® immunoassays (Roche Diagnostics, Mannheim, Germany). These sandwich-immunoassays were developed by applying monoclonal antibodies specifically screened for detection of the respective target. For all biomarkers, the identical plasma sample per blood draw was measured blinded without clinical information being available to laboratory personnel.

Extended Analysis

Sensitivity analyses:

LASSO for data reduction: We used LASSO (least absolute shrinkage and selection operator) to determine the best combination of candidate predictors where forward selection was applied to provide an alternative method for variable selection. In all instances, the LASSO algorithm (Matlab 2019a, The Mathworks Inc, Natick, MA, USA) was applied with 5-fold cross-validation to remove redundant predictors. In the sparsest model, the variables where regularisation had penalised the coefficients by shrinking them to 0 were excluded from the

models and remaining variables with non-zero coefficients were selected (Supplemental Table 1).

Logistic regression: The four clinical characteristics (age, sex, type of AF, LA diameter) and BMP10 were modelled in a logistic regression with forward selection (probability for model entry p = 0.05, removal p = 0.10; outcome = recurrence or no recurrence). The best combination of variables for achieving a significant prediction for recurrent AF consisted of (in order of entry) BMP10, left atrial size, and type of AF (Supplemental Table 2A). This model had an area under the ROC curve (AUC) of 0.688 (95% confidence intervals 0.632, 0.743).

We extended this analysis by considering 11 biomarkers available for this cohort in addition to BMP10 (ANG2, CRP, CA125, ESM1, FGF23, FABP3, GDF15, IGFBP7, IL6, NTproBNP, TnT) in a logistic regression with forward selection. The best combination of variables remained the same as with the Cox regression –BMP10, left atrial size, type of AF, and FGF23 (Supplemental Table 2B).

Additive value of BMP10: We compared models quantified with all 4 clinical characteristics with and without BMP10, and evaluated the additive value of BMP10 (Supplemental Table 3). BMP10 was a significant predictor of recurrent AF in the presence of other variables (HR 1.334, 95%CI 1.142, 1.558). The AUC of the model increased marginally with the addition of BMP10 (Supplemental Figure 4).

Supplemental Figure 1. Uncropped western blots. Western blots are shown for BMP10 blotting (A) and GAPDH blotting (B) of left atrial (LA) and left ventricle (LV) samples from WT and *Pitx2c*$^{+/-}$ mice. The dashed red box corresponds to the cropped bands used in Figure 5C.

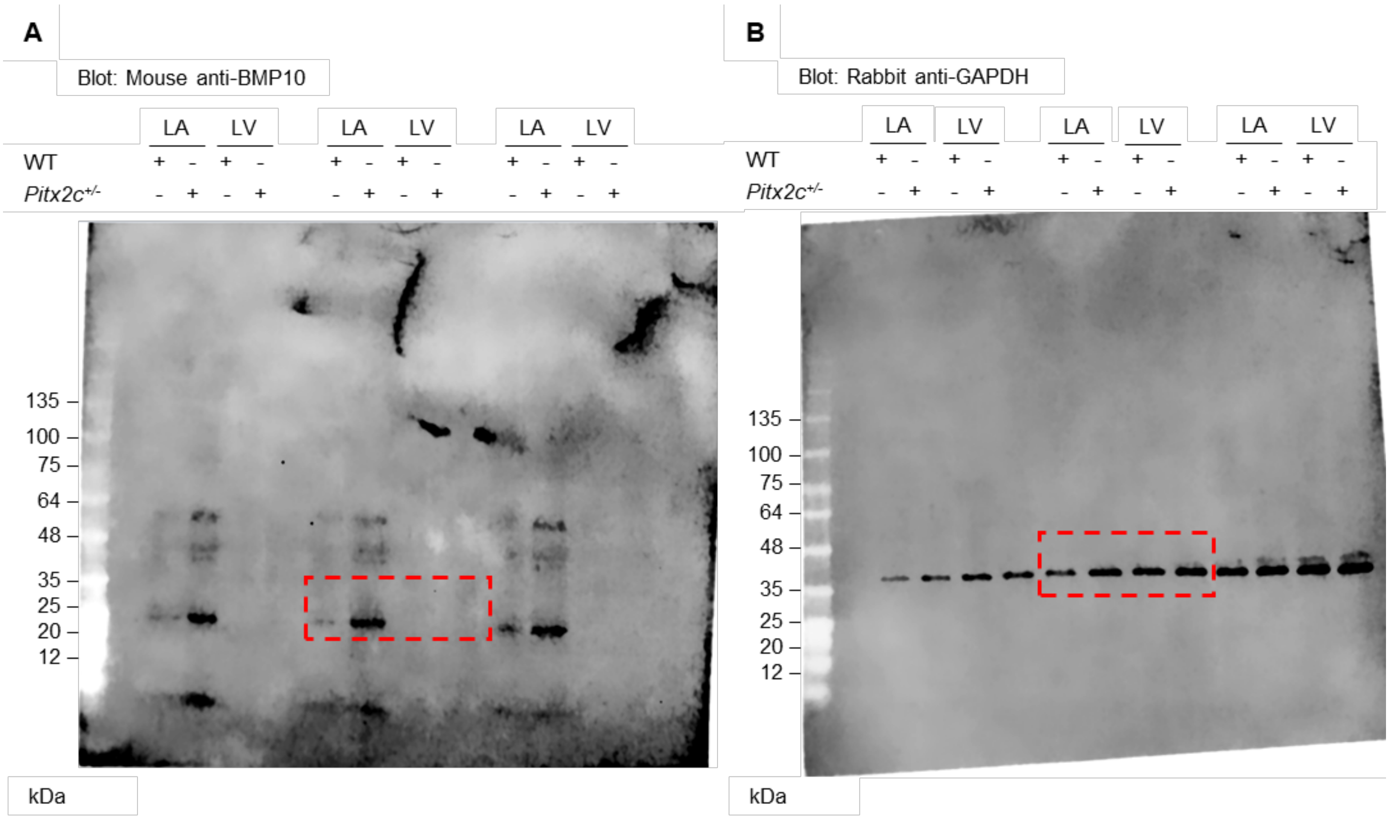

Supplemental Figure 2. Bootstrapping analysis. The multivariate model was corrected for over-optimism by bootstrapping. The bias corrected hazard ratio and confidence intervals were very similar to the fitted model, indicating little to no overfitting. LA, left atrial; BMP10, bone morphogenetic protein 10.

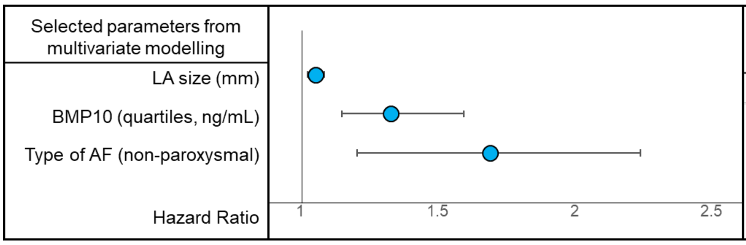

Hazard ratio (HR)	95% confidence interval		B coefficient
	Lower	Upper	
1.055	1.025	1.087	0.054
1.330	1.151	1.595	0.285
1.692	1.208	2.241	0.526
(Baseline cumulative hazard at 1 year)			*0.018*

Supplemental Figure 3. Additional biomarker quantification. Of 12 biomarkers tested, BMP10, FGF23, NTproBNP, FABP3, and GDF15 were the 5 significantly different biomarkers in distinguishing patients with and without recurrent AF. BMP10, FGF23, and to a lesser degree, GDF15, showed a systematic increase of recurrence events by increasing quartiles of biomarkers, indicating their usefulness for risk-stratifying patients. The numbers of patients that experienced recurrent AF in the respective quartiles are shown.
ANG2, angiopoietin 2; BMP10, bone morphogenetic protein 10; CRP, high-sensitivity C-reactive protein; CA125, cancer antigen 125; ESM1, endothelial cell specific molecule 1; FGF23, fibroblast growth factor 23; FABP3, fatty acid binding protein 3; GDF15, growth differentiation factor 15; IGFBP7, insulin like growth factor binding protein 7; IL6, interleukin 6; NTproBNP, N-terminal pro-B-type natriuretic peptide; TnT, high-sensitivity cardiac troponin T.

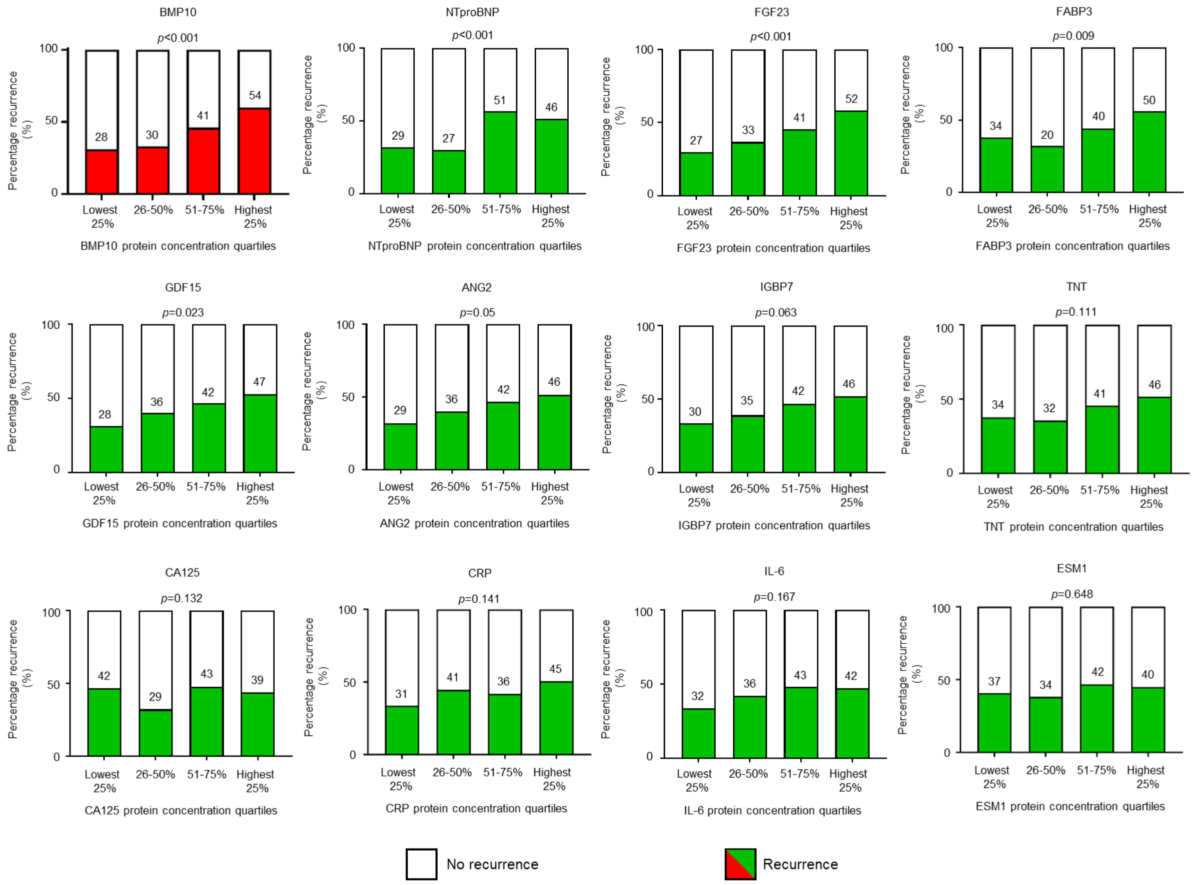

Supplemental Figure 4. Model performance comparison. Area under the ROC (receiver operating characteristic) curve (AUC) and corresponding 95% confidence intervals (CI) of models with and without BMP10. The addition of BMP10 marginally improved the AUC from 0.659 to 0.688.

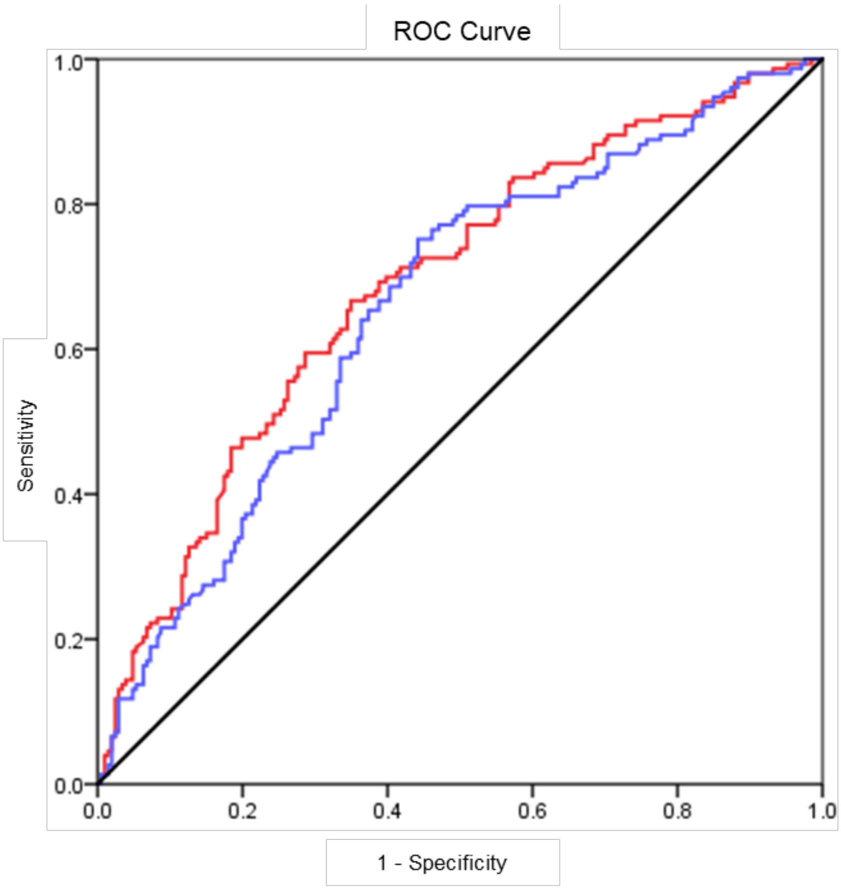

	Legend	AUC (95%CI)
(blue line)	Age, Sex, LA size, type of AF	0.659 (0.602, 0.716)
(red line)	Age, Sex, LA size, type of AF + BMP10	0.688 (0.633, 0.743)

Supplemental Table 1. LASSO for feature selection. Candidate predictors for model fitting identified using the LASSO (least absolute shrinkage and selection operator) algorithm with 5-fold cross-validation. Selected predictors are those in green with non-zero coefficients. The predictor in orange represents the last coefficient (and its corresponding value) to be shrinked to 0. Predictors are same as those selected using forward selection.

Candidate predictors	Coefficient Lambda		
	Clinical predictors + PITX2	Clinical predictors + BMP10	Model with all biomarkers
Age	0	0	0
Sex	0	0	0
Type of AF	0	0.0437	0.0551
Left atrial size	0	0.0051	0.0059
PITX2	-0.2791		
BMP10		0.0591	0.0659
ANG2			0
CRP			0
CA125			0
ESM1			0
FGF23			0
FABP3			0
GDF15			0
IGFBP7			0
IL6			0
NTproBNP			0
TnT			0

Supplemental Table 2. Logistic regression sensitivity analysis.

A

Selected parameter	B coefficient	Hazard ratio (HR)	95% confidence interval	
			Lower	Upper
BMP10 (quartiles, ng/mL)	0.392	1.479	1.209	1.810
LA size (mm)	0.062	1.064	1.024	1.106
Type of AF (non-paroxysmal)	0.602	1.827	1.173	2.844

B

Selected parameter	B coefficient	Hazard ratio (HR)	95% confidence interval	
			Lower	Upper
BMP10 (quartiles, ng/mL)	0.322	1.380	1.116	1.706
LA size (mm)	0.058	1.060	1.020	1.101
Type of AF (non-paroxysmal)	0.539	1.715	1.095	2.687
FGF23 (quartiles, ng/mL)	0.226	1.334	1.142	1.558

Supplemental Table 3. Model outcomes without (a) and with (b) the inclusion of BMP10. Note that BMP10 is significantly associated with recurrent AF.

A

Selected parameter	B coefficient	Odds ratio (OR)	95% confidence interval	
			Lower	Upper
Age (years)	0.001	1.001	0.975	1.028
Sex (female)	-2.276	0.103	0.005	2.151
Age*Sex (interaction)	0.039	1.040	0.993	1.089
LA size (mm)	0.064	1.066	1.026	1.108
Type of AF (non-paroxysmal)	0.676	1.966	1.269	3.046
Constant	-3.513	0.030	-	-

B

Selected parameter	B coefficient	Odds ratio (OR)	95% confidence interval	
			Lower	Upper
Age (years)	-0.005	0.995	0.968	1.023
Sex (female)	-2.516	0.081	0.004	1.766
Age*Sex (interaction)	0.041	1.041	0.994	1.091
LA size (mm)	0.061	1.063	1.023	1.104
Type of AF (non-paroxysmal)	0.609	1.839	1.180	2.867
BMP10 (ng/mL)	0.605	1.832	1.073	3.129
Constant	-4.010	0.018	-	-

References:

1. Syeda F, Holmes AP, Yu TY, Tull S, Kuhlmann SM, Pavlovic D, et al. PITX2 Modulates Atrial Membrane Potential and the Antiarrhythmic Effects of Sodium-Channel Blockers. *Journal of the American College of Cardiology.* 2016;68(17):1881-94.

2. Li L, Tao G, Hill MC, Zhang M, Morikawa Y, and Martin JF. Pitx2 maintains mitochondrial function during regeneration to prevent myocardial fat deposition. *Development.* 2018;145(18).

3. Pfaffl MW. A new mathematical model for relative quantification in real-time RT-PCR. *Nucleic Acids Res.* 2001;29(9):e45.
